# An appressorium membrane protein, Pams1, controls infection structure maturation and virulence via maintaining endosomal stability in the rice blast fungus

**DOI:** 10.3389/fpls.2022.955254

**Published:** 2022-09-09

**Authors:** Jing Wang, Qing Wang, Pengyun Huang, Yingmin Qu, Zhicheng Huang, Huan Wang, Xiao-Hong Liu, Fu-Cheng Lin, Jianping Lu

**Affiliations:** ^1^State Key Laboratory for Managing Biotic and Chemical Threats to the Quality and Safety of Agro-products, College of Life Sciences, Zhejiang University, Hangzhou, China; ^2^State Key Laboratory for Managing Biotic and Chemical Threats to the Quality and Safety of Agro-products, Institute of Plant Protection and Microbiology, Zhejiang Academy of Agricultural Sciences, Hangzhou, China; ^3^Institute of Biotechnology, Zhejiang University, Hangzhou, China

**Keywords:** *Magnaporthe oryzae*, appressorium formation, endosome, cAMP, pathogenicity, cell death, cAMP-PKA, endolysosome

## Abstract

The rice blast fungus *Magnaporthe oryzae* spores differentiate and mature into functional appressoria by sensing the host surface signals. Environmental stimuli are transduced into cells through internalization during appressorium formation, such as in the cAMP-PKA pathway. Here, we describe a novel contribution to how appressoria mature on the surface of a leaf, and its connection to endosomes and the cAMP-PKA pathway. An appressorium membrane-specific protein, Pams1, is required for maintaining endosomal structure, appressorium maturation, and virulence in *M. oryzae*. During appressorium development, Pams1 was translocated from the cell membrane to the endosomal membrane. Deletion of *PAMS1* led to the formation of two types of abnormal appressoria after 8 h post inoculation (hpi): melanized type I had a reduced virulence, while pale type II was dead. Before 8 hpi, Δ*pams1* formed appressoria that were similar to those of the wild type. After 8 hpi, the appressoria of Δ*pams1* was differentiated into two types: (1) the cell walls of type I appressoria were melanized, endosomes were larger, and had a different distribution from the wild type and (2) Type II appressoria gradually stopped melanization and began to die. The organelles, including the nucleus, endosomes, mitochondria, and endoplasmic reticula, were degraded, leaving only autophagic body-like vesicles in type II appressoria. The addition of exogenous cAMP to Δ*pams1* led to the formation of a greater proportion of type I appressoria and a smaller proportion of type II appressoria. Thus, defects in endosomal structure and the cAMP-PKA pathway are among the causes of the defective appressorium maturation and virulence of Δ*pams1*.

## Introduction

Plant pathogenic fungi continue to threaten global food security (Fisher et al., 2012). *Magnaporthe oryzae* (synonym *Pyricularia oryzae*), the rice blast disease pathogen, causes serious damage to the production of cereal crops, such as rice and wheat (Talbot, 2003; Inoue et al., 2017). *M. oryzae* infects plants via an appressorium, a specialized infection structure (Howard and Valent, 1996). Spores of *M. oryzae* fall on the surface of the plant, germinate, produce germ tubes, and differentiate into appressoria (Jiang et al., 2018). Before differentiating into appressoria, conidial germ tubes sense surface hardness (Liu H. et al., 2007), hydrophobic signals (Lee and Dean, 1994), and chemical molecules, for example, cutin monomers (Lee and Dean, 1993; Skamnioti and Gurr, 2007). A mature and functional appressorium is necessary for the infection of a plant by *M. oryzae* (Chumley and Valent, 1990; deJong et al., 1997; Dagdas et al., 2012). The developmental process of an appressorium can be divided into three stages: (1) In the initiation or differentiation stage, germ tube hooking and swelling occurs, followed by bulbous apex formation (Wilson and Talbot, 2009); (2) The maturation stage consists of melanization of cell walls, the influx of three-celled spore inclusion into a unicellular appressorium, generation of high turgor pressure, and septin ring formation inside the appressorium (Wilson and Talbot, 2009; Jiang et al., 2018); (3) Finally, the penetration stage takes place with the infection peg emerging from an appressorium (Dagdas et al., 2012). During appressorium formation, spore cells die through autophagy and ferroptosis, and intracellular storage is degraded and transported into the appressorium to build new cellular structures and synthesize solutes such as glycerol (Veneault-Fourrey et al., 2006; Liu X. H. et al., 2007; Shen et al., 2020). Blocking autophagy inhibits spore cell death and degradation and reduces appressorial turgor pressure and virulence in *M. oryzae* (Liu X. H. et al., 2007).

The cAMP-PKA signaling pathway controls the recognition of surface signals during the initiation of an appressorium (Wilson and Talbot, 2009; Li Y. et al., 2017; Jiang et al., 2018). Deletion of *CPKA*, a catalytic subunit of Protein Kinase A (PKA), resulted in smaller appressoria and reduced virulence in *M. oryzae* (Xu and Hamer, 1996; Xu et al., 1997), and Δ*cpkA*Δ*cpk2*, in which two catalytic subunits of PKA were deleted, completely failed to produce appressoria on hydrophobic surfaces (Selvaraj et al., 2017). Pth11 (a G-protein-coupled receptor), Gα (the α-subunit of heterotrimeric G proteins), Mac1 (an adenylate cyclase), and Rgs1 (the regulator of G protein signaling 1) finely regulate cAMP signaling through endosomes (Zhang et al., 2011; Ramanujam et al., 2013; Kou and Naqvi, 2016; Kou et al., 2017). The endocytosis of the plasma membrane is triggered by the activation of cell surface receptors, and the activated receptors along with their bound extracellular material enter the cell interior via endosomes (Scott et al., 2014). Early endosomes are highly dynamic organelles that fuse with themselves or other types of vesicles. The membrane and its activated cell surface receptors return to the plasma membrane via recycling endosomes, while some late endosomes and multivesicular bodies (MVBs) fuse with lysosomes or vacuoles to degrade their contents (Scott et al., 2014). In *M. oryzae*, the receptor protein Pth11 enters endosomes from the plasma membrane through endocytosis, and several cAMP-PKA signaling pathway proteins such as Mac1, Rgs1, and Gα are located on endosomes (Zhang et al., 2011; Ramanujam et al., 2013; Kou and Naqvi, 2016). Impaired endocytosis reduces appressorium formation and virulence in *M. oryzae* (Zhang et al., 2011; Ramanujam et al., 2013).

The Pmk1 kinase signaling pathway controls appressorium formation and penetration (Jiang et al., 2018; Ryder et al., 2019), and the Mps1 kinase signaling pathway regulates appressorium penetration (Xu et al., 1998; Jiang et al., 2018). Glucose-ABL1-TOR signaling and cell cycle arrest finely regulate appressorium morphogenesis (Marroquin-Guzman and Wilson, 2015; Marroquin-Guzman et al., 2017). Pmk1-dependent Mst12 and Hox7 transcriptional network control appressorium formation and plant penetration (Oses-Ruiz et al., 2021). Deletion of a transcription factor *HOX7* disabled formation of mature appressoria in *M. oryzae* (Kim et al., 2009; Oses-Ruiz et al., 2021). In a large-scale functional study of 47 Cys2-His2 transcription factors of *M. oryzae*, we found that Vrf1 is another key transcription factor controlling appressorium maturation and deletion of *VRF1* prevents the incipient appressorium from maturing (Cao et al., 2016). Δ*vrf1* formed an appressorium with similar morphology as the wild type within 6 h post inoculation (hpi). After that point in time, however, the mutant’s appressorium did not develop into a functional hemispherical appressorium, but gradually became a hypha-like structure (Cao et al., 2016). Our recent study showed that Δ*vrf1* and Δ*hox7* have a similar mutant phenotype during appressorium formation and that the double knockout mutant of *VRF1* and *HOX7* (Δ*vrf1*Δ*hox7*) also has a similar mutant phenotype to Δ*vrf1* and Δ*hox7* during appressorium formation (Huang et al., 2022). Δ*vrf1*, Δ*hox7*, and Δ*vrf1*Δ*hox7* failed to form mature appressoria on hydrophobic surfaces but formed hyphal-like structures. Since the virulence of *M. oryzae* requires mature functional appressoria, the study of the appressorium maturation mechanism can lead to an in-depth understanding of the interaction mechanism between the fungus and rice.

In this study, we identified a novel appressorium membrane-specific protein, Pams1, that is necessary for appressorium morphogenesis and fungal virulence on rice via endosomes and the cAMP-PKA signaling pathway during appressorium maturation in *M. oryzae*.

## Materials and methods

### Fungal strains, growth conditions, and DNA/RNA manipulation

*Magnaporthe oryzae* strain 70–15 and its derivatives (Supplementary Table 1) were cultured on a complete medium (CM) at 25°C under a 16-h light/8-h dark cycle (Talbot et al., 1993). DNA and RNA manipulations were conducted following standard procedures as previously reported (Sambrook and Russell, 2001; Lu et al., 2014). Total RNAs were extracted from fungal samples using the RNeasy Plant Mini Kit (Qiagen, United States). Real-Time Quantitative Reverse Transcription PCR (qRT-PCR) was performed to obtain gene expression levels from three to five biological replicates using *40S* and α-*ACTIN* as reference genes. Expression levels were calculated using the 2^–Δ*CT*^ method after normalization to the two reference genes. The PCR primers used in this study are listed in Supplementary Table 2.

### Deletion and complementation of targeted genes in *Magnaporthe oryzae*

A *PAMS1* deletion mutant was generated using a method described previously (Lu et al., 2014; Yan et al., 2019). The gene-deletion cassette was built in pKO3B using a hygromycin B phosphotransferase gene (*HPH*) as a selection gene and transformed into *M. oryzae* strains via *Agrobacterium tumefaciens*-mediated transformation (ATMT) according to a reported method (Lu et al., 2014; Yan et al., 2019). Transformants were grown on selection media containing 0.5 μM 5-fluoro-2′-deoxyuridine (F2dU) and 200 μg ml^–1^ hygromycin B. The null mutants were confirmed by PCR to confirm a deleted gene using β-*TUBULIN* DNA as a positive control and another PCR to detect a targeted recombinational DNA sequence of the resistance gene (Supplementary Figure 1). The copies of transformed gene-deletion cassettes in mutants were identified by quantitative PCR (qPCR) (Lu et al., 2014), and in all the mutants verified in Supplementary Figure 1, the resistance gene was inserted in a single copy (Supplementary Table 3). The mutants were purified by monoconidiation for further experiments. Δ*pams1* was complemented with the native copy of *PAMS1* that was cloned into the *Eco*RI-*Xba*I site of pKD5 containing a sulfonylurea resistance gene (*SUR*) using a fusion enzyme (Vazyme, China) (Li et al., 2012). The gene-rescued transformants were identified on selection plates supplemented with 100 μg ml^–1^ chlorimuron-ethyl and confirmed at the mRNA level by reverse transcription PCR (RT-PCR) (Supplementary Figure 1).

### Phenotypic analysis

Mycelial growth and conidiation of strains were assayed on CM at 25°C for 8 days. For conidial germination (at 4 hpi) and appressorium formation assays (at 4–24 hpi), 25 μl droplets of spore suspensions (1 × 10^5^ conidia ml^–1^) were placed on plastic coverslips (Thermo Scientific, United States) or hydrophobic polyvinyl chloride (PVC) films and incubated at 22°C in the dark. Appressorium turgor pressure was evaluated through incipient cytorrhysis (cell collapse) assays as described previously (Howard et al., 1991). At 24–48 hpi, appressoria were incubated in a series of glycerol solutions (0.5–2.0 M) for 5 min and then the collapsed appressoria were counted under a microscope. Ten mM 8-Bromoadenosine 3′,5′-cyclic monophosphate sodium salt (8-Bromo-cAMP, Santa Cruz Biotechnology, United States) were used in the appressorium formation assay. The experiments were performed three times with three or five replicates each time. For conidial germination and appressorium formation assays, 110–200 spores or appressoria were counted per replicate.

### Plant infection assays

Barley (*Hordeum vulgare* cv. ZJ-8) or rice (*Oryza sativa* cv. CO-39) seeds were sterilized with sodium hypochlorite solution for 3–4 min, rinsed repeatedly with water, and then planted in soil at 25°C for 8 days (barley) or 14 days (3–4 leaf stage for rice). For virulence assays with leaf explants of barley, 5-mm mycelium blocks were inoculated on leaves and cultured at 25°C for 4 days. For the spraying assay on rice, 2-week-old rice seedlings (*n* ≥ 17) were inoculated by spraying with 2.5 ml conidial suspensions (5 × 10^4^ conidia ml^–1^) containing 0.2% (w/v) gelatin. The disease severity was assessed within a 5-cm section of the infected leaf exhibiting the most serious disease lesions in each seedling at 7 dpi (days post inoculation) until full disease symptoms on the wild type became apparent (Cao et al., 2016). For observations of the invasive process, spore suspensions (1 × 10^5^ conidia ml^–1^) were inoculated on barley leaves and rice leaf sheaths. Two sets of each plant species were incubated at 25°C, one set for each of two incubation times, 24 and 48 h. Host cells infected by appressoria were observed under a microscope after each incubation time (Cao et al., 2018). The experiments were repeated three times.

### Observation of cytoplasm and glycogen

To visualize surviving cytoplasm, cells were stained with 2 μg ml^–1^ fluorescein diacetate (FDA) (Sigma-Aldrich, United States) for 5–10 min or glycogen inside cytoplasm was stained with solutions containing 60 mg ml^–1^ KI and 10 mg ml^–1^ I_2_ for 2 min (Thines et al., 2000). To label endolysosomes and vacuoles, 7-amino-4-chloromethylcoumarin (CMAC, Invitrogen, United States) solution (10 μM final concentration) was added to spore suspensions, and the spores were inoculated on hydrophobic borosilicate glass coverslips and incubated for 24 hpi. Samples were observed under a fluorescence microscope (Nikon Eclipse Ni, Japan) or a Leica icc50 W microscope (Leica, Germany). The experiments were performed three times with three replicates each time, and 110–200 spores or appressoria were counted per replicate.

### Electron microscope observation

Samples were prepared following a standard procedure (Lu et al., 2009). Spore suspensions were inoculated on barley leaves and cultivated for 18 h at 25°C. And the appressoria were covered with a thin layer of 3.5% agar. The appressoria along with leaves were fixed with 2.5% glutaraldehyde in 0.1 M phosphate buffer (pH 7.3) overnight at 4°C, fixed with 1% osmium acid in 0.1 M phosphate buffer (pH 7.3) for 1 h, dehydrated with ethanol and acetone, and embedded in Spurr resin. The ultrathin sections were stained with lead citrate and uranyl acetate solution for 5–10 min and observed with a transmission electron microscope (H-7650, Hitachi, Japan). Five to 10 slices per sample were observed. The experiments were performed two times, and three independent biological samples were prepared each time.

### Observation of fluorescence fusion proteins

To build the *GFP-PAMS1* infusion gene, the native *PAMS1* (containing its own promoter and terminator) along with *GFP* (that was inserted into the site between amino acids 50 and 51 of Pams1) were fused into the *Eco*RI-*Xba*I site of pKD5-GFP containing *SUR* (Li et al., 2012). The *GFP-PAMS1* expression vector was transformed into Δ*pams1* via ATMT. pKD3-mCherry is a vector containing *mCherry* and *BAR* (a bialaphos resistance gene) instead of the *GFP* and *SUR* found in pKD5-GFP, and pKD10-mCherry is a vector with the *H3* promoter of pKD3-mCherry replaced with the β-*TUBULIN* promoter. To visualize endosomes, nuclei, ER, mitochondria, and lipid droplets, one of each of the five vectors, pKD3-mCherry-Rab5A (*RAB5A* was fused into the C-terminus of mCherry), pKD10-H_2_B-mCherry (*H_2_B* fused into the N-terminus of mCherry of pKD10-mCherry), pKD8-Spf1-GFP (Qu et al., 2020), pKD8-Atp1-GFP (Shi et al., 2018), and pKD8-Cap20-GFP (Cao et al., 2018) were transformed into one of each of the wild type, Δ*pams1*, and Δ*pams1*^*GFP–PAMS*1^ (Δ*pams1* expressing *GFP-PAMS1*) cultures. Appressoria were induced on hydrophobic borosilicate glass coverslips (Thermo Scientific, United States) and observed under a fluorescence microscope and a laser scanning confocal microscope (FV3000, Olympus, Japan). The observation of each sample was repeated at least three times, more than 100 spores or appressoria were observed each time, and dozens of pictures were taken.

### Quantification of glycerol contents

Equal volumes of spore suspensions (5 × 10^5^ conidia ml^–1^) of Δ*pams1* and the wild type were separately inoculated onto PVC films at 25°C in the dark. At 24 hpi, the glycerol contents in appressoria and culture solutions were each measured using a glycerol assay kit (MAK117, Sigma-Aldrich, United States). Glycerol contents are shown in nmol per 10^6^ appressoria. The experiments were performed three times with three replicates each time.

### Bioinformatics analysis

Transmembrane helices in Pams1 were predicted by the TMHMM Server v. 2.0^1^ and NCBI conserved domain data^2^. Image analysis software (FV31S-SW, FV31S-DT, or Image J) was used to show fluorescence photos or fluorescence intensity distribution plots.

### Statistical analysis

Each value shown for each group is the mean ± SD (standard deviation). All experiments were repeated independently at least three times. The *P*-value was calculated using an unpaired two-tailed Student’s *t*-test in GraphPad Prism 8. *P*-values < 0.05 were considered significant, while *P*-values > 0.05 were considered non-significant.

## Results

### Identification of an appressorium-specific membrane protein, Pams1, in *Magnaporthe oryzae*

**FIGURE 1:**
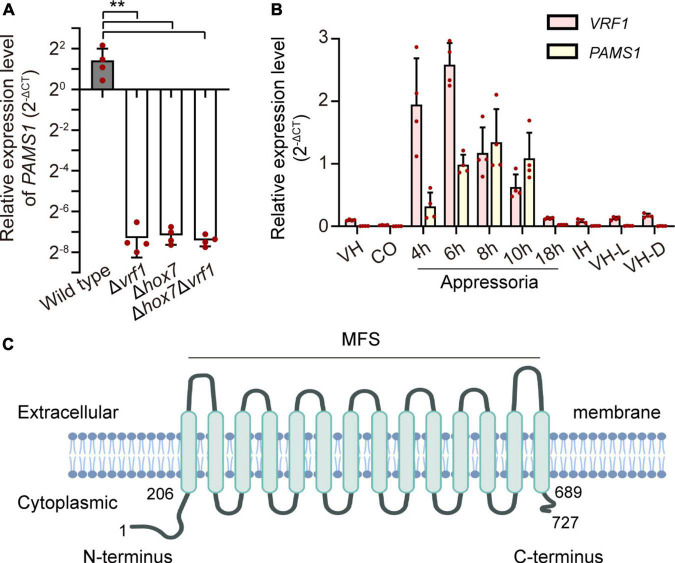
Identification of an appressorium membrane protein Pams1 in *M. oryzae*. **(A)** Relative expression levels of *PAMS1* in 5-hpi appressoria of the wild type and Δ*vrf1*, Δ*hox7*, and Δ*hox71*Δ*vrf1* (*n* = 4). **P < 0.01. **(B)** Relative transcript levels of *PAMS1* and *VRF1* in the wild type (*n* = 4). VH, vegetative hyphae in CM. CO, conidia. 4–18 h, appressoria sampled at 4–18 hpi. IH, invasive hyphae in barley at 2 dpi. VH-L or VH-D, aerial hyphae cultured under light or darkness, respectively. *40S* and α-*ACTIN* are used as reference genes in panels **(A,B)**. **(C)** Transmembrane helices in Pams1 were predicted by the TMHMM software and NCBI conserved domain data.

**FIGURE 2:**
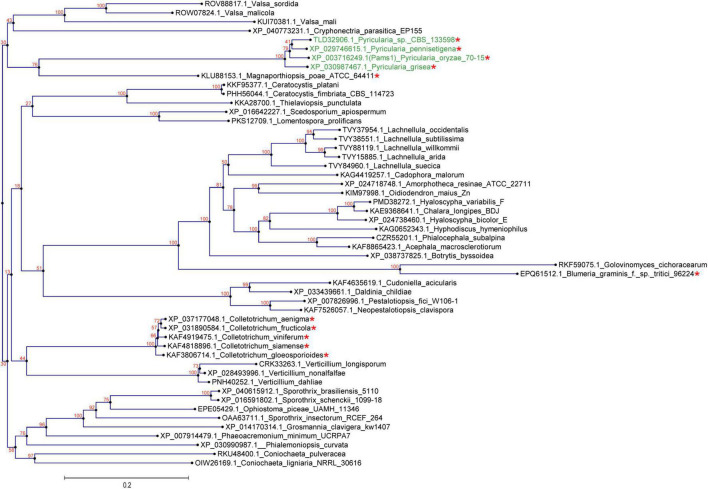
Homologs of Pams1 are present in other fungi. Phylogenetic tree (constructed by the neighbor-joining method) of Pams1 homologs. The proteins used to build this tree were selected from the top 100 homologs of Pams1 with *E*-values < 9.1E-176 obtained by BLAST search of the NCBI protein database. The top five species with the smallest *E*-values in a genus were first selected, and then one protein from each species with the smallest *E*-value was selected. *M. oryzae* (syn. *Pyricularia oryzae*) and related three species of *Pyricularia* (*Magnaporthe*-like sexual morphs) are indicated in green. Asterisks indicate species that form appressoria or appressoria-like structures. The numbers at branch nodes are the bootstrap values. The scale bar indicates amino acid substitutions per site.

**FIGURE 3:**
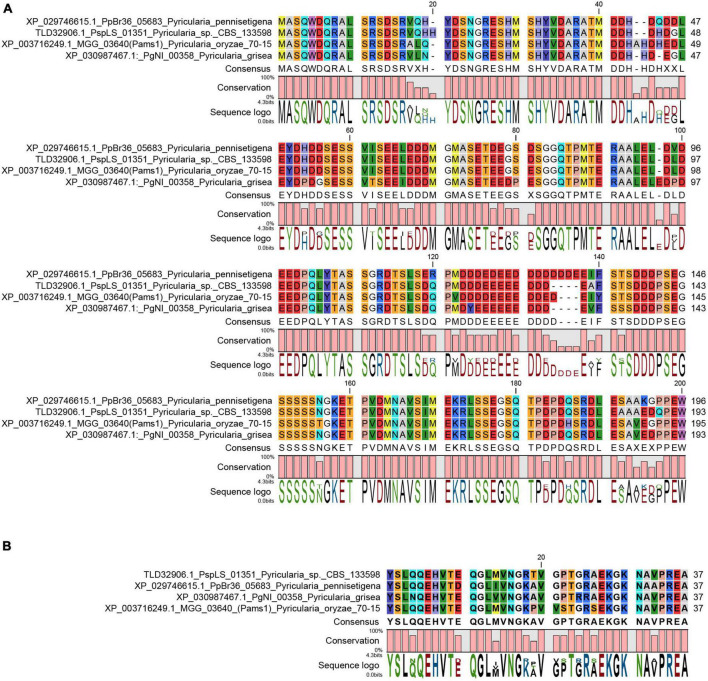
N-terminal and C-terminal peptide sequences of Pams1 were only present in *Magnaporthe* species. **(A,B)** Alignment of Pams1-N **(A)** and Pams1-C **(B)** homologs (*E*-value < 1.0) by BLAST search using the respective Pams1-N (1–195 aa) or Pams1-C (691–727 aa) peptide sequences in NCBI.

In our previous study, appressoria formed by a *VRF1*-deleted mutant cannot develop into mature and functional appressoria (Cao et al., 2016). A total of 913 genes were downregulated by more than four times (FDR < 0.01) in the appressoria of Δ*vrf1* than in that of the wild type at 5 hpi (Cao et al., 2016). The downregulation of some key genes in Δ*vrf1* is one of the reasons for the mutant phenotype. To identify genes that may be involved in appressorium maturation, we knocked out 78 of these downregulated genes and found a mutant 11D9 with defects in appressorium maturation, in which *MGG_03640* (XP_003716249.1) was deleted (Supplementary Figure 1; Supplementary Table 3). Since the study described below suggested MGG_03640 as a membrane protein functioning specifically at the appressorium stage, here, MGG_03640 was named Pams1 (*Pyricularia* appressorium membrane-specific protein 1). *PAMS1* was downregulated 423.2-, 385.5-, and 457.7-fold in the 5-hpi appressoria of Δ*vrf1*, Δ*hox7*, and Δ*hox7*Δ*vrf1*, respectively (Figure 1A). Quantitative RT-PCR analysis showed that *PAMS1* expression was high in appressoria of 4–10 hpi and very low in hyphae and spores, and this temporal distribution pattern was similar to the mRNA expression pattern of *VRF1* and *HOX7* (Cao et al., 2016; Huang et al., 2022), suggesting that the expression of *PAMS1* is developmental stage-dependent (Figure 1B). TMHMM server predicted that Pams1 encodes a membrane protein with 14 transmembrane regions that constitute an major facilitator superfamily (MFS) domain (Figure 1C). Homologs of Pams1, mainly MFS domain, are present in other fungi (Figure 2). However, the homologous peptides in a 195-amino acid sequence of the N-terminus (1–195 aa, Pams1-N) or a 37-amino acid peptide of the C-terminus (691–727 aa, Pams1-C) of Pams1 are only present in *Magnaporthe* species (Figures 3A,B).

### Pams1 is required for virulence, but not for growth and conidiation

**FIGURE 4:**
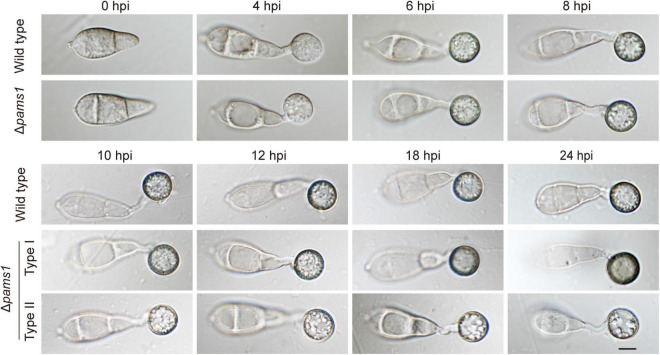
Characteristics of Δ*pams1* in appressorium formation. Appressoria formed by Δ*pams1* and the wild type at 0, 4, 6, 8, 10, 12, 18, and 24 hpi. Δ*pams1* formed two types of abnormal appressoria (types I and II) after 10 hpi. Scale bar, 5 μm.

**FIGURE 5:**
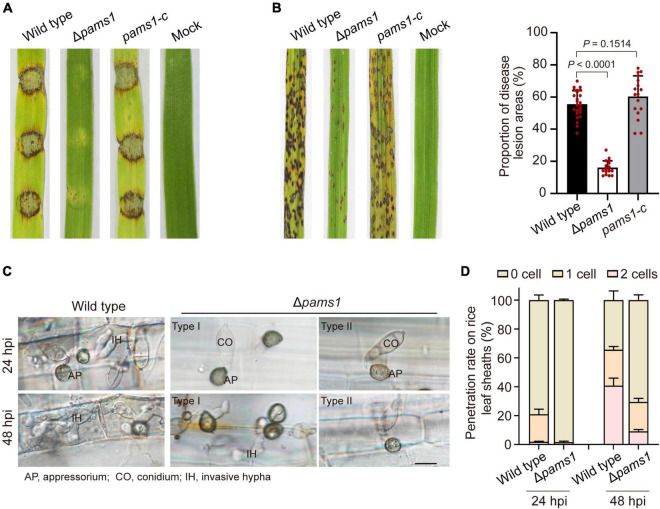
*PAMS1* is required for fungal virulence. **(A)** The 5-mm mycelial blocks were inoculated on leaf explants of barley. Images were taken at 4 dpi. **(B)** Left, virulence assays on rice by Δ*pams1*, its complementation strain *pams1-c* and the wild type. A suspension of 5 × 10^4^ conidia per ml was sprayed onto seedlings of the rice cultivar CO-39. Images were taken at 7 dpi. Right, dot plot showing lesion areas calculated from a 5-cm zone of the infected rice leaf exhibiting the most serious disease lesions in each seedling. **(C)** Host penetration by appressoria and invasive hyphal growth on rice leaf sheaths at 24 and 48 hpi. Type II abnormal appressoria of Δ*pams1* were unable to invade rice tissue, and the invasion of type I abnormal appressoria into host tissue was delayed until 48 hpi. Scale bar, 10 μm. **(D)** Percentages of appressoria with invasive hyphae that infected 0 to 3 rice cells (*n* = 3) at 24 and 48 hpi. At least 150 appressoria were counted per replicate.

We analyzed the phenotype of Δ*pams1* in development and virulence. The phenotypic analysis of Δ*pams1* showed that its colony growth, sporulation, spore germination, and appressorium formation rates were comparable to those of the wild type (Supplementary Figure 2). However, the Δ*pams1* appressoria formed on plastic coverslips gradually underwent structural deformation after 10 hpi. Finally, two types of abnormal appressoria were formed at 24 hpi (Figure 4). When inoculated on barley leaves with mycelial blocks, the virulence of Δ*pams1* was greatly reduced (Figure 5A). When sprayed as spore suspensions on rice seedlings, Δ*pams1* showed significantly reduced virulence as compared to the wild type (Figure 5B). In rice leaf sheath assays, Δ*pams1* showed reduced host penetration and invasive growth (Figures 5C,D). In Δ*pams1*, melanized type I appressoria displayed a delayed penetration and invasive growth, and pale type II appressoria could not penetrate rice cells (Figure 5C).

### Pams1 is required for appressorium maturation

**FIGURE 6:**
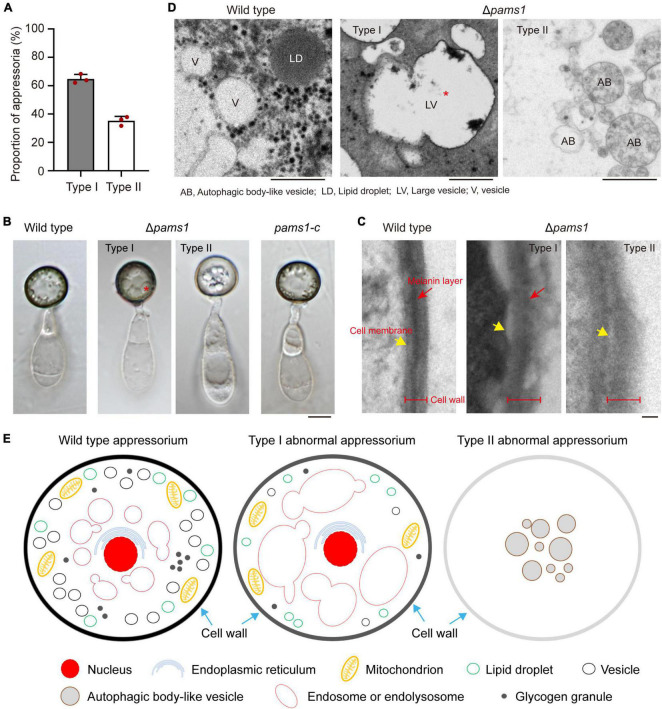
Δ*pams1* formed two types of abnormal appressoria. **(A)** Proportions between the two types of abnormal appressoria on plastic coverslips at 24 hpi (*n* = 3). At least 200 appressoria were counted per replicate. **(B)** Appressoria of Δ*pams1*, its complementation strain *pams1-c*, and the wild type at 24 hpi. Δ*pams1* formed two types of abnormal appressoria (types I and II). Scale bar, 5 μm. **(C)** Ultrastructure of the appressorial cell wall. Scale bar, 100 nm. **(D)** Ultrastructure of appressorial vesicles. Scale bars, 1 μm. **(E)** Schematic diagram of three types of appressoria based on microstructure, ultrastructure, and fluorescently labeled organelles (in Figures 6–8, 10, 11) after 18 hpi. The red stars refer to large vesicles in panels **(B,D)** are endosomes or endolysosomes.

To reveal the structural differences between the two types of appressoria, we carefully assayed the mature appressorium structure of Δ*pams1* (Figure 6). Δ*pams1* formed 64.8% melanized type I and 35.2% pale type II abnormal appressoria on plastic coverslips at 24 hpi (Figure 6A). There was no difference in shape between the two types of appressoria, but the cell wall color and internal structure were different (Figures 4, 6B). One (type I) had a black cell wall and fewer but larger-sized intracellular vesicles at 24 hpi relative to the wild type. The other (type II) had a grayish cell wall and fewer intracellular vesicles at 24 hpi when compared with the wild type (Figures 4, 6B). The ultrastructure showed that the density of the melanin layer in the cell wall decreased from the wild type (normal) to type I and type II (abnormal) appressoria at 18 hpi (Figure 6C). Compared with the wild type, the vesicles in type I appressoria were large and branched, and the cytoplasm was deeply stained, while the cytoplasm of type II appressoria was nearly transparent (Figure 6D). The vesicles in type II appressoria were autophagic body-like vesicles (Figures 6D,E).

**FIGURE 7:**
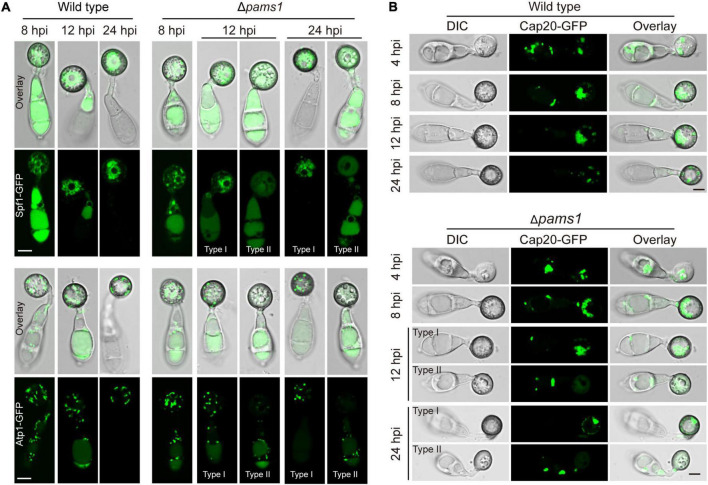
Endoplasmic reticula, mitochondria, and lipid droplets in Δ*pams1*’s appressoria. **(A)** Distribution of Spf1-GFP-marked endoplasmic reticula and Atp1-GFP-marked mitochondria in appressoria of the wild type and Δ*pams1* (types I and II abnormal appressoria) at 8, 12, and 24 hpi. **(B)** Distributions of lipid droplets in spores and appressoria at 4, 8, 12, and 24 hpi. Lipid droplets were marked by Cap20-GFP. Scale bar, 5 μm.

**FIGURE 8:**
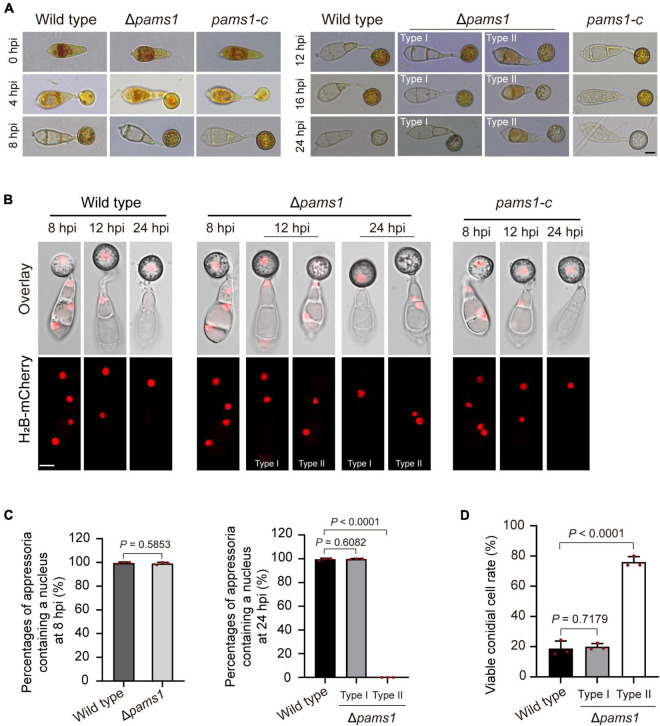
Glycogen granules and nuclei in Δ*pams1*’s appressoria and viable conidial cells in spores that formed appressoria. **(A)** Distributions of glycogen granules in spores and appressoria at 0, 4, 8, 12, 16, and 24 hpi. Glycogen granules were stained by KI/I_2_ solution. Glycogen granules in spore cells that formed type II abnormal appressoria were not fully mobilized into appressoria at 24 hpi. **(B)** Distribution of H_2_B-mCherry-marked nuclei in appressoria of the wild type, complementation strain *pams1-c*, and Δ*pams1* (types I and II abnormal appressoria) at 8, 12, and 24 hpi. **(A,B)** Scale bars, 5 μm. **(C)** Percentages of appressoria containing a nucleus at 8 and 24 hpi (*n* = 3). At least 150 appressoria were counted per replicate. **(D)** Percentages of viable conidial cells in spores that formed appressoria at 24 hpi (*n* = 3). The spores were inoculated on plastic coverslips for 24 hpi and stained with FDA. At least 240 conidial cells were counted per replicate.

To understand differences in organelle dynamics during appressorium formation between the wild type and Δ*pams1*, we observed cellular organelles in the germinated spores and developing appressoria on hydrophobic borosilicate glass coverslips by labeling the organelles (Figures 7, 8). At 8 hpi, Δ*pams1* formed only one type of appressoria that contained endoplasmic reticula, mitochondria, and lipid droplets, which was similar to that in the wild type (Figure 7). At 12 and 24 hpi, the wild type and melanized type I abnormal appressoria had endoplasmic reticula, mitochondria, and lipid droplets in their appressorial cells, while pale type II abnormal appressoria lost normally structured endoplasmic reticula, mitochondria, and lipid droplets (Figure 7). At 24 hpi, the endoplasmic reticula, mitochondria, and lipid droplets in spore cells of the wild type and Δ*pams1* that formed type I abnormal appressoria had fully degraded or/and migrated into appressoria, while those in the mutant spores that formed type II abnormal appressoria were still present in spore cells (Figure 7). Glycogen granules were transferred from spore cells to an appressorium in a similar manner between the wild type and Δ*pams1* that formed type I abnormal appressoria during appressorium formation, while glycogen granules were retained in spore cells of Δ*pams1* that formed type II abnormal appressoria at 24 hpi (Figure 8A). At 8 hpi, the spores and appressoria of the wild type and Δ*pams1* had one nucleus per cell. At 12 and 24 hpi, the wild type and Δ*pams1* that formed melanized type I abnormal appressoria had one nucleus in each appressorium, whereas pale type II abnormal appressoria of Δ*pams1* lost their nuclei (Figures 8B,C). At 24 hpi, the spores of Δ*pams1* that formed type II abnormal appressoria contained nuclei (Figure 8B). The degradation of the nucleus and other organelles in type II abnormal appressorium after 12 hpi suggests that the appressorium had died.

To further identify the viability of spore cells of Δ*pams1* at 24 hpi, we stained the germinated spores on hydrophobic borosilicate glass coverslips with FDA. The results displayed that the cytoplasm of 76.06% of the spore cells that formed pale type II malformed appressoria showed green fluorescence (Figure 8D; Supplementary Figure 3), indicating that the spore cells were still viable and the cytoplasm in spore cells had not completely migrated into appressoria. However, the cytoplasm in most spore cells that formed melanized type I malformed appressoria fully mobilized into the appressorial cells, similar to that observed in the wild type. In the pale type II malformed appressoria of Δ*pams1*, many mitochondria, endoplasmic reticula, glycogen granules, lipid droplets, and nuclei were not degraded and migrated from the spore cells into the appressoria (Figures 7, 8).

### The appressorial cell wall structure of Δ*pams1* is severely defective

**FIGURE 9:**
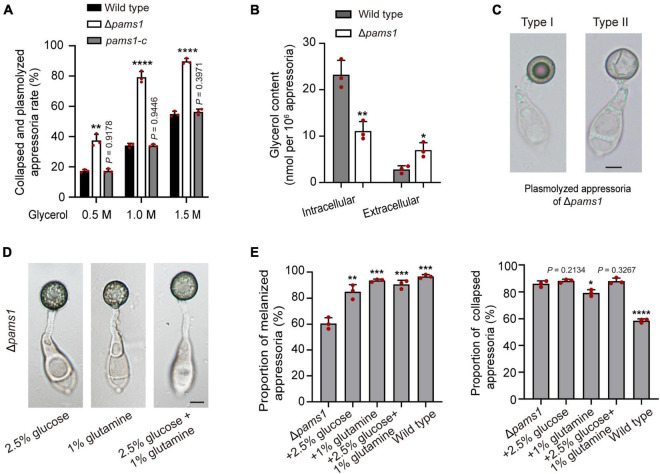
Roles of Pams1 in appressorium cell walls. **(A)** Incipient cytorrhysis assays for appressorium turgor pressure (*n* = 3). The appressoria at 24 hpi were incubated in 0.5, 1.0, or 1.5 M of glycerol solutions, and the collapsed and plasmolyzed appressoria were counted under a microscope. At least 140 appressoria were counted per replicate. **(B)** Intracellular and extracellular glycerol contents of appressoria formed by the wild type and Δ*pams1* on hydrophobic polyvinyl chloride (PVC) films at 24 hpi (*n* = 3). **(C)** Plasmolyzed appressoria (types I and II) formed by Δ*pams1* at 24 hpi and incubated in a 1.5 M glycerol solution. **(D)** Appressoria were formed by Δ*pams1* after the addition of exogenous glucose and/or glutamine at 24 hpi. **(E)** Addition of exogenous 2.5% glucose and/or 1% glutamine obviously increased the number of melanized appressoria (Left panel) but did not greatly increase appressorium turgor pressure (Right panel) in Δ*pams1* (*n* = 3). Appressorium turgor pressure was assayed in a 1.5 M glycerol solution. At least 200 appressoria at 24 hpi were counted per replicate. Unpaired two-tailed Student’s *t*-test was used for comparisons with non-treated Δ*pams1* in panel **(E)** or with the wild type in panels **(A,B)**. *P < 0.05, **P < 0.01, ***P < 0.001, ****P < 0.0001. **(C,D)** Scale bars, 5 μm.

The ultrastructure of appressorial cell walls showed that the melanin layer was destroyed in Δ*pams1* (Figure 6C). The melanin layer of the cell wall prevented the escape of high concentrations of glycerol accumulated in an appressorium, resulting in huge intracellular turgor pressure (Chumley and Valent, 1990; deJong et al., 1997; Ryder et al., 2019). To evaluate the effect of a defective melanin layer on appressorial turgor, incipient cytorrhysis assays were conducted to determine turgor pressure of appressoria of Δ*pams1* in a series of glycerol solutions of varying molarity (Ryder et al., 2019). The proportions of total cell plasmolysis and collapse of Δ*pams1* appressoria formed on plastic coverslips at 24 hpi were much higher than that of the wild type (Figure 9A), indicating that the turgor pressure in mutant appressoria was lower than that in the wild type. Analysis of the glycerol content at 24 hpi showed that the intracellular glycerol content in the Δ*pams1* appressoria incubated on hydrophobic PVC films was lower than that in the wild type appressoria, while the amount of extracellular glycerol leaking into the incubation solution from the mutant appressoria was much higher than that from the wild type appressoria (Figure 9B). In addition, the ability of mutant appressorium to adhere to hydrophobic surfaces was not different from that of the wild type. When treated with higher concentrations of a glycerol solution, many appressorial protoplasts of Δ*pams1* exhibited plasmolysis (Supplementary Figure 4A). Among them, the cell membranes of some melanized type I appressoria were completely separated from the cell wall, forming a spherical protoplast (Figure 9C). The evidence indicates that the appressorial cell wall structure of Δ*pams1* appressoria is severely defective.

### Exogenous glucose and amino acids partially abolish the Δ*pams1*’s defect in appressorium maturation

To understand whether the death of Δ*pams1*’s type II abnormal appressoria is caused by appressorial nutrient deficiency resulting from disruption of spore cell death, we observed the effect of exogenous nutrients on the appressorium maturation of Δ*pams1*. After adding 2.5% glucose, 1% glutamine, or both simultaneously into spore suspensions, the proportion of melanized appressoria formed by Δ*pams1* was significantly higher than that without treatment (Figures 9D,E; Supplementary Figure 4B). These melanized appressoria were similar in appearance to the wild type appressoria (Figure 9D; Supplementary Figure 4C). However, the cytoplasm and organelles of spores were still not fully mobilized into the melanized appressoria (Figure 9D; Supplementary Figure 4C), suggesting that the formation of pale type II abnormal appressoria in Δ*pams1* is related to the unavailability of carbon and nitrogen sources in the mutant appressoria. Under 1.5 M glycerol solution, the percentage of collapsed or plasmolyzed appressoria in Δ*pams1* with the addition of glucose or both glucose and glutamine was still similar to that without treatment (Figure 9E; Supplementary Figure 4C), indicating that the melanin layer structure of mutant appressoria was still defective.

### Loss of Pams1 results in fusion and enlargement of endosomes in appressoria

**FIGURE 10:**
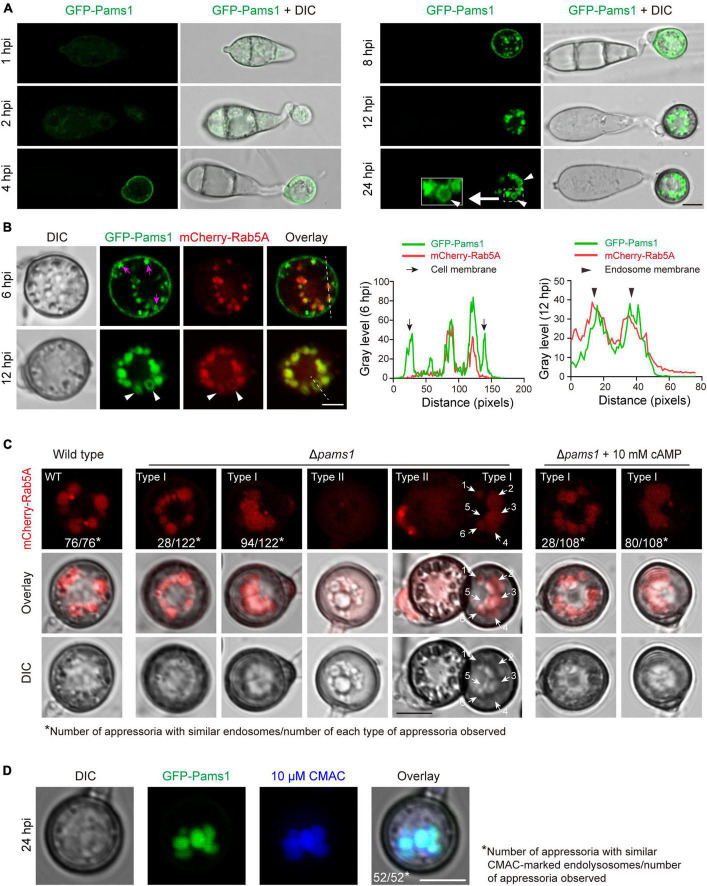
Pams1 is required for the morphology and distribution pattern of endosomes in appressoria. **(A)** Localization of GFP-Pams1 in spores and developing appressoria at 1–24 hpi. The strain is the complementation strain of Δ*pams1* constructed by introducing *GFP-PAMS1*. **(B)** Left, co-localization of GFP-Pams1 and the endosome marker mCherry-Rab5A in appressoria at 6 and 12 hpi. Right, line-scan graphs showing GFP-Pams1 and mCherry-Rab5A fluorescence density in transverse sections of several endosomes. Pams1 and Rab5A are co-localized in the endosomal membrane. Magenta arrowheads indicate vesicles linked with the cell membrane. The strain is Δ*pams1* expressing both *GFP-PAMS1* and *mCherry-RAB5A*. White triangular arrowheads indicate endosome membranes **(A,B)**. **(C)** Endosomes marked by mCherry-Rab5A in the appressoria of the wild type and Δ*pams1* at 24 hpi. White arrows with numbers refer to six endosomes. **(D)** Endosomes or endolysosomes were stained by 10 μM CMAC in appressoria of the complementation strain of Δ*pams1* constructed by introducing *GFP-PAMS1*. Spore suspensions were added with CMAC solution (final concentration 10 μM), inoculated on hydrophobic borosilicate glass coverslips, and cultured for 24 hpi. Scale bars, 5 μm.

To explore where and how Pams1 functions, we observed the spatiotemporal localization of Pams1 during appressorium maturation. The *GFP-PAMS1* fusion gene under the control of its native promoter was transformed into Δ*pams1* and could complement the mutant phenotype of Δ*pams1*. GFP-Pams1 expression mainly occurred in appressoria (Figure 10A). Pams1 was initially distributed in membranes of cells and small vesicles at 4–6 hpi, then it was gradually transferred to mid-sized vesicle membranes after 6 hpi, and ultimately coalesced in large-vesicle membranes after 12 hpi (Figure 10A). GFP-Pams1 was co-localized with the endosome marker mCherry-Rab5A in endosome membranes, which indicates that the Pams1-containing intracellular vesicles were endosomes (Figure 10B).

**FIGURE 11:**
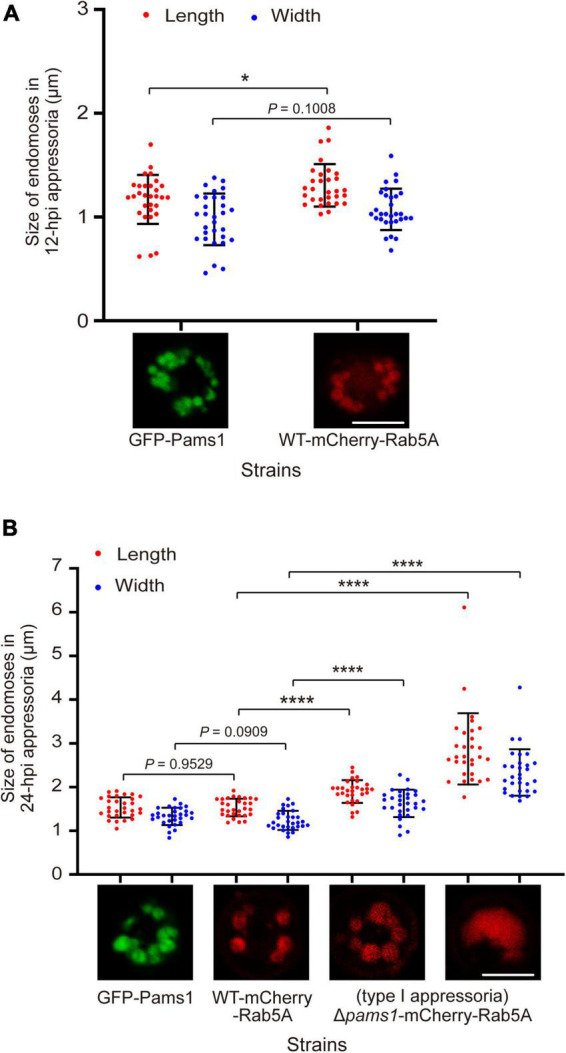
Pams1 prevents excessive fusion of endosomes during appressorium maturation in *M. oryzae*. **(A)** Size of 12-hpi appressorial endosomes. **(B)** Size of 24-hpi appressorial endosomes. The largest three endosomes in each appressorium were selected to measure their size, and a total of 10 appressoria were measured. The strains used here are as follows: GFP-Pams1 (the complementation strain of Δ*pams1* expressing *GFP-PAMS1*), WT-mCherry-Rab5A (the wild type strain expressing *mCherry-RAB5A*), and Δ*pams1*-mCherry-Rab5A (Δ*pams1* expressing *mCherry-RAB5A*). Spore suspensions were inoculated on hydrophobic borosilicate glass coverslips and incubated for 12 or 24 hpi. The GFP-Pams1 and WT-mCherry-Rab5A strains formed normal appressoria, and the Δ*pams1*-mCherry-Rab5A strain formed two types of abnormal appressoria, in which only endosomes or endolysosomes in type I appressoria were measured. *p < 0.05, ****p < 0.0001. Scale bars, 5 μm.

The morphology and distribution patterns of endosomes in Δ*pams1* were different from those of the wild type. Relative to the wild type, the endosomes of type I abnormal appressoria in Δ*pams1* were merged into larger vacuoles at 24 hpi. In the wild type, endosomes were circularly arranged in appressoria at 24 hpi. In Δ*pams1*, however, the endosomes of type I abnormal appressoria were arranged circularly or semi-circularly, whereas the endosomes of type II appressoria were disintegrated (Figure 10C). The expression of mCherry-Rab5A slightly increased endosomal size at 12 hpi but not at 24 hpi (Figures 11A,B). However, the size of endosomes of type I abnormal appressoria in Δ*pams1* was greatly larger than that of the wild type (Figures 10C, 11), meaning that deletion of *PAMS1* resulted in increased fusion between endosomes or with other vesicles or vacuoles. After the addition of a vacuolar dye CMAC to the spore suspensions, CMAC co-localized with GFP-Pams1 in appressorial endosomes at 24 hpi (Figure 10D). Thus, these appressorial endosomes at 24 hpi were endolysosomes. Relative to the control (Figure 10A: 24 hpi), however, 24 h of CMAC treatment promoted fusion of endosomes in appressoria (Figure 10D). In the type I appressoria of Δ*pams1* at 24 hpi, the large vacuoles observed under differential interference contrast (DIC) were co-localized with the fluorescence signal of mCherry-Rab5A (Figure 10C), suggesting that these large vacuoles were merged endolysosomes. These merged large endolysosomes were also seen in Figures 6B,D, marked with red stars in the type I appressoria of Δ*pams1* at 24 hpi.

### Pams1 is involved in the cAMP-PKA pathway during appressorium maturation

**FIGURE 12:**
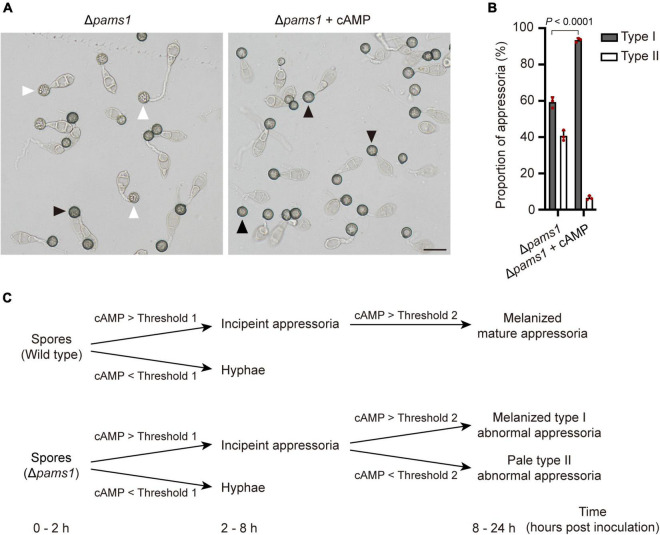
Addition of exogenous cAMP increased the proportion of melanized type I abnormal appressoria formed by Δ*pams1* on plastic coverslips. **(A)** Appressoria formed by Δ*pams1* on plastic coverslips after treatment with 10 mM cAMP at 24 hpi. Black arrows indicate type I abnormal appressoria, and white arrows indicate type II abnormal appressoria. Scale bar, 20 μm. **(B)** Proportions of types I and II abnormal appressoria formed by Δ*pams1* on plastic coverslips treated with 10 mM cAMP. The wild type did not form abnormal appressoria on plastic coverslips. At least 110 appressoria were counted per replicate. **(C)** Schematic illustration of how intracellular cAMP levels affect the differentiation of spores into incipient appressoria and then into mature appressoria in Δ*pams1*. On a membrane surface, if the intracellular cAMP content is higher than threshold 1, the germinated spore of Δ*pams1* differentiates into an incipient appressorium or it differentiates into a hypha; if the intracellular cAMP of the incipient appressorium is higher than threshold 2, the appressorium continues to develop into a melanized type I abnormal appressorium, otherwise it develops into a pale type II abnormal appressorium.

*Magnaporthe oryzae* senses plant surface signals and differentiate them into appressoria via the cAMP-PKA and Pmk1 MAP kinase pathways (Jiang et al., 2018). Among them, the cAMP-PKA signal is transmitted into the cell through the internalization of the cell membrane to form endosomes (Li et al., 2019). To check if the cAMP-PKA pathway was disturbed in Δ*pams1*, we tested the effect of exogenous cAMP on appressorium maturation. After adding 10 mM of cAMP analogs (8-Bromo-cAMP) into spore suspensions, Δ*pams1* formed a higher proportion of melanized type I abnormal appressoria on plastic coverslips at 24 hpi, as compared to the result in the absence of cAMP treatment (Figures 12A,B). Furthermore, the addition of exogenous cAMP did not eliminate the mutant’s defect in endosomal fusion (Figure 10C).

## Discussion

Rice blasts can cause the most serious fungal epidemics in rice production (Talbot, 2003; Inoue et al., 2017). Appressorium formation, penetration, and infective growth mechanisms of *M. oryzae* have long been the foci of much academic research (Howard and Valent, 1996; Talbot, 2003; Jiang et al., 2018; Ryder et al., 2019; Huang et al., 2022). In this study, we identified an appressorium-specific membrane protein, Pams1, which is required for appressorium maturation and virulence in *M. oryzae*. *PAMS1* was highly expressed in the wild type appressoria; however, it was greatly downregulated in the incipient appressoria of Δ*vrf1*, Δ*hox7*, and Δ*hox7*Δ*vrf1*. Both Vrf1 and Hox7 are transcription factors regulating appressorium maturation in *M. oryzae* (Kim et al., 2009; Cao et al., 2016; Oses-Ruiz et al., 2021; Huang et al., 2022).

The Δ*pams1* mutant showed a reduced virulence on barley and rice. This reduced virulence is owing to its decreased appressorial penetration into plant cuticles. Deletion of *PAMS1* resulted in the formation of two types of abnormal appressoria which were different from those formed by the wild type in structure. These two types of abnormal appressoria have not been reported in *M. oryzae*. Appressorium formation is divided into three stages: differentiation, maturation, and penetration (Dagdas et al., 2012; Inoue et al., 2017). The time point at which the Δ*pams1* mutant differentiated into two types of appressoria (10 hpi) was slightly later than the time when the normal appressoria of the wild type completed expansion and started cell wall melanization (before 8 hpi) (Howard and Valent, 1996; Dean, 1997). Comparing the appressorium morphogenesis and maturation process in the wild type and Δ*pams1*, 8–10 hpi was found as the time point for an appressorium to transit from the differentiation stage to the mature stage.

Abnormal endosome structure and the impaired cAMP-PKA signaling pathway during appressorium maturation are two reasons why Δ*pams1* forms two types of abnormal appressoria. Several large vesicles were observed in the DIC pictures and the electron microscope pictures of the Δ*pams1* appressoria at 18 or 24 hpi. These large branched vesicles have structural features of late endosomes and endolysosomes (Kou and Naqvi, 2016). They were co-localized with the endosomal marker protein Rab5A, suggesting that these large vesicles of Δ*pams1* observed by electron or light microscopy were abnormal endosomes or endolysosomes. In the appressoria of the wild type strain, endosomes were relatively small and not easily distinguishable from other types of vesicles in DIC pictures.

In *M. oryzae*, spores germinate and sense the induction signals from rice surfaces and synthesize cAMP, which in turn activates PKA kinases and appressorium formation (Jiang et al., 2018). Component members of the cAMP-PKA pathway, such as Pth11, Gα, Rgs1, and Mac1, receive hydrophobic signals and chemical molecules on the plant surface and are internalized from the cell membrane (Zhang et al., 2011; Ramanujam et al., 2013; Kou and Naqvi, 2016; Li et al., 2019). These proteins in the signaling pathway are localized in the endosomal membrane. Endocytosis is required for receptor internalization, signaling activation, and appressorium formation (Li X. et al., 2017). Pams1 was specifically expressed in the appressoria of *M. oryzae*. At 4–12 hpi, Pams1 was gradually translocated from the cell membrane to the endosomal membrane by endocytosis. Pams1 inhibited excessive fusion between endosomes or with vacuoles during appressorium maturation. The endosome structure of the appressorium was abnormal in Δ*pams1*. Relative to the wild type, Δ*pams1* formed larger endosomes or endolysosomes at 24-hpi appressoria. We speculate that due to structural defects in the endosomes or endolysosomes, environmental stimuli sensed by the maturing appressorium were not properly transmitted into the cell, resulting in dysfunction of the cAMP-PKA pathway and insufficient cAMP synthesis in Δ*pams1*. In Δ*pams1*, appressorium differentiation was unaffected, but the addition of exogenous cAMP promoted the mutant to form a higher proportion of melanized type I appressoria, suggesting that a fraction of the maturing appressoria did not contain enough cAMP to mature. In other words, cAMP synthesis was defective in these appressoria. The degree of reduction in synthesized cAMP content varied among the maturing appressoria of Δ*pams1*. There may be a threshold of cAMP content below which the incipient appressorium cannot continue to develop into a mature appressorium, but instead enters a process of cell death. However, the threshold for this cAMP content remains unknown. Before 8 hpi, the appressorial cell walls underwent preliminary melanization. After 8 hpi, the appressoria with intracellular cAMP content above the threshold continued to develop and mature, with continued melanization of the cell walls and transportation of the cytoplasm in spore cells to appressorial cells. In contrast, development and cell wall melanization gradually stopped in the appressoria with lower intracellular cAMP content than the threshold. As a result, at 24 hpi, the Δ*pams1* mutant formed two types of abnormal appressoria: melanized type I appressoria and pale type II appressoria, in which type II appressoria were dead. This means that the formation of two types of appressoria reflects the difference in the degree of reduction in intracellular cAMP levels among the incipient appressoria of Δ*pams1* (Figure 12C).

In Δ*pams1*, the pale type II appressoria were not viable after 12 hpi, and vesicles in these appressoria were autophagic body-like vesicles (Liu X. H. et al., 2007). During the appressorium formation of *M. oryzae*, autophagy and ferroptosis are involved in the death process of spore cells (Veneault-Fourrey et al., 2006; Shen et al., 2020). After autophagy or ferroptosis was blocked, the germinated spore cells continued to survive, and their intracellular contents could not be degraded and transported into an appressorium (Veneault-Fourrey et al., 2006; Shen et al., 2020), which is similar to the spore cells that formed pale type II appressoria in Δ*pams1*. However, loss of autophagy and ferroptosis did not cause the death of appressoria themselves (Veneault-Fourrey et al., 2006; Lu et al., 2009; Shen et al., 2020), which is different from that in the pale type II appressoria of Δ*pams1*. Autophagic body-like vesicles appeared in type II appressoria of Δ*pams1*, indicating that the activation of autophagy is involved in the death process of type II appressoria. The addition of an exogenous nitrogen source or carbon source could prevent Δ*pams1* from forming pale type II appressorium, which means that it reverses the death process or inhibits autophagy of the appressorium. This is consistent with the basic mechanism of autophagy, that is, starvation induces autophagy (Kuma et al., 2004). Defects in the TOR signaling pathway led to the germinated spores forming long tubes and delayed differentiation of irregular appressoria (Marroquin-Guzman et al., 2017; Sun et al., 2019). These irregular appressoria are morphologically different from those formed by Δ*pams1*. The appressoria of Δ*pams1* were as normal as the wild type before 10 hpi. In mammals, cAMP can promote or inhibit autophagy, depending on the cell type (Grisan et al., 2021). The addition of exogenous cAMP promoted conidial death and prevented appressorial death in Δ*pams1*, implying that cAMP promotes autophagy in germinated conidial cells and inhibits autophagy in developing appressoria in *M. oryzae*. However, the relationship between Pams1 and the cAMP-PKA pathway, TOR signaling, autophagy, or cell cycle arrest needs to be carefully studied in future.

In summary, we identified the biological function of an appressorium-specifically expressed protein, Pams1. The localization of Pams1 is translocated from the cell membrane to the endosomal membrane before the maturation of an appressorium. Pams1 is involved in the maintenance of the morphology and distribution pattern of endosomes, as well as the function of the cAMP-PKA pathway in a maturing appressorium. *PAMS1* is required for appressorium maturation and pathogenicity, but not for colony growth, sporulation, spore germination, and appressorium differentiation in *M. oryzae*.

## Data availability statement

The original contributions presented in this study are included in the article/Supplementary material, further inquiries can be directed to the corresponding author.

## Author contributions

JL and JW conceived and designed the study, analyzed the data, and wrote the manuscript. JW, QW, PH, ZH, YQ, and HW performed the experiments with phenotypical and biochemical assays. F-CL, X-HL, and JL contributed to the reagents, plant, and fungal materials. JW, QW, and PH collected the data. All authors contributed to the article and approved the submitted version.

## Footnotes

1
https://services.healthtech.dtu.dk

2
www.ncbi.nlm.nih.gov/Structure/cdd/wrpsb.cgi
