# Supplementary material for: An appressorium membrane protein, Pams1, controls infection structure maturation and virulence via maintaining endosomal stability in the rice blast fungus
Source: Front Plant Sci. 2022 Sep 9;13:955254. doi: 10.3389/fpls.2022.955254 (PMC9500233; doi:10.3389/fpls.2022.955254)
Supplement: Supplementary file 1 [file Table_1.docx]

**Supplementary Table S1 Strains used in this study.**

| Strains | Genotype description | Reference |
| --- | --- | --- |
| 70-15 | Wild-type | Chao et al., 1991 |
| Δ*pams1* | *PAMS1* deletion mutant of 70-15 | This study |
| *pams1-c* | *PAMS1* rescued strain of Δ*pams1* | This study |
| Δ*vrf1* | *VRF1* deletion mutant of 70-15 | Cao et al., 2016 |
| Δ*hox7* | *HOX7* deletion mutant of 70-15 | Huang et al., 2022 |
| Δ*vrf1*Δ*hox7* | *VRF1 and HOX7 deletion mutant of 70-15* | Huang et al., 2022 |
| WT-Spf1-GFP | *SPF1*-GFP transformant of 70-15 | This study |
| Δ*pams1*-Spf1-GFP | *SPF1*-GFP transformant of Δ*pams1* | This study |
| WT-Atp1-GFP | ATP1-*GFP* transformant of 70-15 | This study |
| Δ*pams1*-Atp1-GFP | ATP1-*GFP* transformant of Δ*pams1* | This study |
| WT-Cap20-GFP | *CAP20-GFP* transformant of 70-15 | This study |
| Δ*pams1*-Cap20-GFP | *CAP20-GFP* transformant of Δ*pams1* | This study |
| WT-H_2_B-mCherry | *H_2_B-mCherry* transformant of 70-15 | This study |
| Δ*pams1*-H_2_B-mCherry | *H_2_B-mCherry* transformant of Δ*pams1* | This study |
| GFP-Pams1 | *GFP-PAMS1* transformant of Δ*pams1* | This study |
| GFP-Pams1/mCherry-Rab5A | Both *GFP-PAMS1* and *mCherry-RAB5A* transformant of Δ*pams1* | This study |
| WT-mCherry-Rab5A | *mCherry-RAB5A* transformant of 70-15 | This study |
| Δ*pams1*-mCherry-Rab5A | *mCherry-RAB5A* transformant of Δ*pams1* | This study |
| *pams1-c-*H_2_B-mCherry | *H_2_B-mCherry* transformant of *pams1-c* | This study |

Chao, C.C.T., and Ellingboe, A.H. (1991). Selection for mating competence in *Magnaporthe-grisea* pathogenic to rice. *CAN J BOT.* **69**, 2130-2134.

Cao, H., Huang, P., Zhang, L., Shi, Y., Sun, D., Yan, Y., Liu, X. Dong, B., Chen, G., Snyder, J.H. *et al.* (2016). Characterization of 47 Cys2 -His2 zinc finger proteins required for the development and pathogenicity of the rice blast fungus *Magnaporthe oryzae*. *New Phytol.* **211**, 1035-1051.

Huang, P.Y., Wang, J., Li, Y., Wang, Q., Huang, Z., Qian, H., Liu., X-H., Lin, F-C., Lu., J. (2022). Transcription factors Vrf1 and Hox7 coordinately regulate appressorium maturation in the rice blast fungus *Magnaporthe oryzae*. *Microbiol Res*. **263**, 127141.
