# Supplementary material for: An appressorium membrane protein, Pams1, controls infection structure maturation and virulence via maintaining endosomal stability in the rice blast fungus
Source: Front Plant Sci. 2022 Sep 9;13:955254. doi: 10.3389/fpls.2022.955254 (PMC9500233; doi:10.3389/fpls.2022.955254)
Supplement: Supplementary file 2 [file Table_2.docx]

**Supplementary TABLE S2** Primers used in this study.

| 1. Primers used in qPCR | |
| --- | --- |
| VRF1-qF | GCAAGCGACCATACAAGTG |
| VRF1-qR | AGAGTCTGAAGTGCATTCGTC |
| PAMS1-qF | CCATGATCAGCAAGGGAATCT |
| PAMS1-qR | GTAGAACTTGACAGCCTGGTAG |
| 40S-qF | ACAAGCTCAAGACCCTCGTC |
| 40S-qR | GGTGGTGATGGTGAAGCAG |
| Actin-F | ACAATGGTTCGGGTATGTGC |
| Actin-R | CGACAATGGACGGGAAGAC |
|  |  |
| 2. Primers used to build knockout and complementation strains | |
| PAMS1-upF (5f) | GAGGCTAACTGACACTCTAGAAGCATATTTAGGACCGCATACC |
| PAMS1-upR (5r) | CATTCATTGTTGACCTCCACTAGTGCAGGCTATGAAACAGGA |
| PAMS1-dnF (3f) | GGGCAAAGGAATAGAGTAGATGGTGCATGATTGGAGCGTAGTA |
| PAMS1-dnR (3r) | GAGCTGTACAAGTAAAAGCTTGGAGTTCCTAGACCAGTTTGTG |
| HPH-F | TAGTGGAGGTCAACAATGAATG |
| HPH-R | CTATTCCTTTGCCCTCGGACGA |
| PAMS1-S-F (kof) | GGTGGCTCGTTGATCTTTACA |
| PAMS1-S-R (kor) | CCCACTGTTTGACCGAGAATAG |
| PAMS1-LF (p1) | CATCAGTCTAGACTAGTTGAGG |
| HPH-CKR (p2) | GGGCGAACTTAAGAAGGTATGA |
| Tbl-gF | TTCCGCGCTGTCACCGTTCC |
| Tbl-gR | GGGCCTCCTCCTCGTACTCCTCTT |
| qtub-F | ACAACTTCGTCTTCGGTCAG |
| qtub-R | GTGATCTGGAAACCCTGGAG |
| qHPH-F | ATGTCCTGCGGGTAAATAGC |
| qHPH-R | GATGCAATAGGTCAGGCTCTC |
| PAMS1c-F | TTCACAATCACTAGTGAATTCCCGTACCTGGGAATACTCAAC |
| PAMS1c-R | TTACTGCAGGTCGACTCTAGACGGTCAAAGATGTAGGCAGTAT |
| qPAMS1-RT-F | CAGGCATAGTCGTTGGTCTC |
| qPAMS1-RT-R | ATGTCTTGGGCATGAGTAGC |
| qTbl-RT-F | TTCCGCGCTGTCACCGTTCC |
| qTbl-RT-R | GGGCCTCCTCCTCGTACTCCTCTT |
|  |  |
| 3. Primers used to build fluorescent-fusion protein vectors | |
| PKD5-PAMS1-F | TTCACAATCACTAGTGAATTCCCGTACCTGGGAATACTCAAC |
| GFP-PAMS1-50-R | CTCCTCGCCCTTGCTCACCATTTCCAAGTCTTCATGATCGTGAGC |
| GFP-PAMS1-51-F | GGCATGGACGAGCTGTACAAGTACGACCATGACGACTCGGAATC |
| PKD5-PAMS-R | TTACTGCAGGTCGACTCTAGACGGTCAAAGATGTAGGCAGTAT |
| GFP-F | ATGGTGAGCAAGGGCGAGGAG |
| GFP-R | CTTGTACAGCTCGTCCATGCC |
| RED-PAMS1-50-R | GACGTTCTCGGAGGAGGCCATTTCCAAGTCTTCATGATCGTGAGC |
| RED-PAMS1-51-F | GGCCGCCACCACCTGTTCCTG TACGACCATGACGACTCGGAATC |
| RED-F | ATGGCCTCCTCCGAGAACGTCAT |
| RED-R | CAGGAACAGGTGGTGGCGG |
| PKD3-RAB5A-F | GTCACCGAGATTTAGGAATTCCTTTCTGCCCTTGGTCTCTT |
| Promoter-RAB5A-R | CTCCTCGCCCTTGCTCACCATGGCGGGAATTCACCTCGATCG |
| mCherry-RAB5A-F | CCGCCACTCCACCGGCGGCGGGCGGCGGCGGCGGCGGCATGGCCGACGGAGCCGCCAA |
| PKD3-RAB5A-R | TTACTGCAGGTCGACTCTAGAGAGAATTAGTCGAGTAGCGTC |
| PKD10-H2B-mCherry-F | TAGTGGAGGTCAACAATGAATG |
| PKD10-H2B-mCherry-R | CTATTCCTTTGCCCTCGGACGA |
