# Supplementary material for: An appressorium membrane protein, Pams1, controls infection structure maturation and virulence via maintaining endosomal stability in the rice blast fungus
Source: Front Plant Sci. 2022 Sep 9;13:955254. doi: 10.3389/fpls.2022.955254 (PMC9500233; doi:10.3389/fpls.2022.955254)
Supplement: Supplementary file 3 [file Table_3.docx]

**Supplementary TABLE S3** Copy number identification of the resistant gene inserted in the mutant genome by qPCR.

| Mutant No | Copy numbers of a resistant gene *HPH*^a^ | Insertion event |
| --- | --- | --- |
| Δ*pams1*-1 | 0.96 | Single^b^ |
| Δ*pams1*-2 | 1.07 | Single |
| Δ*pams1*-3 | 1.00 | Single |
| Δ*pams1*-4 | 0.89 | Single |

^a^ Copies of *HPH* in the mutant genome were quantified by qPCR after normalization with the *β-TUBULIN* gene.

^b^ “Single” represents the targeted gene deletion event without ectopic insertion.
