# Supplementary material for: An appressorium membrane protein, Pams1, controls infection structure maturation and virulence via maintaining endosomal stability in the rice blast fungus
Source: Front Plant Sci. 2022 Sep 9;13:955254. doi: 10.3389/fpls.2022.955254 (PMC9500233; doi:10.3389/fpls.2022.955254)
Supplement: Supplementary file 4 [file Image_1.pdf]

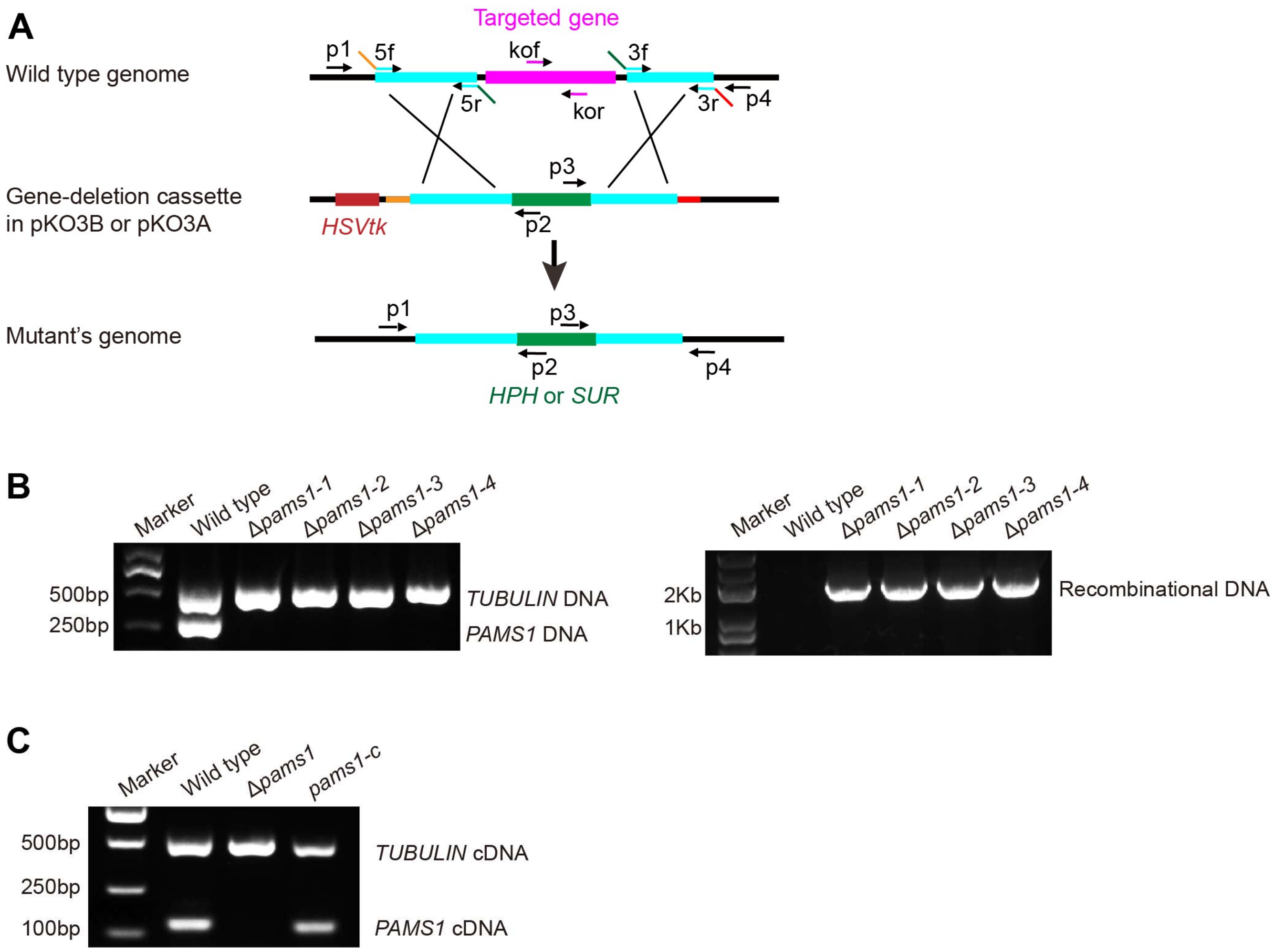

Supplementary FIGURE S1 Knockout and complementation of *PAMS1* in *Magnaporthe oryzae* strain 70-15. (**A**) Targeted gene deletion strategy. Two pairs of primers (5f/5r and 3f/3r) were used to clone the respective left and right flanking fragments of the targeted genes. Primer set kof/kor were used to clone a fragment of the targeted deletion gene in transformants. Primer sets p1/p2 or p3/p4 were used to clone the recombinational DNA fragments in mutants. pKO3A and pKO3B are two vectors containing *HSVtk* (a suicide gene). (**B**) *PAMS1*-deleted events in the wild type 70-15 were confirmed at the DNA level by PCR for both *PAMS1* (amplified by primers kof/kor) and the positive control of *β-TUBULIN* DNA (left panel) and by PCR for the recombinational DNA event using primer sets p1/p2 or p3/p4 (right panel). (**C**) Complementation of Δ*pams1* by *PAMS1* (*pams1-c*). The complement of *PAMS1* was confirmed at the RNA level by RT-PCR using *β-TUBULIN* as a control.

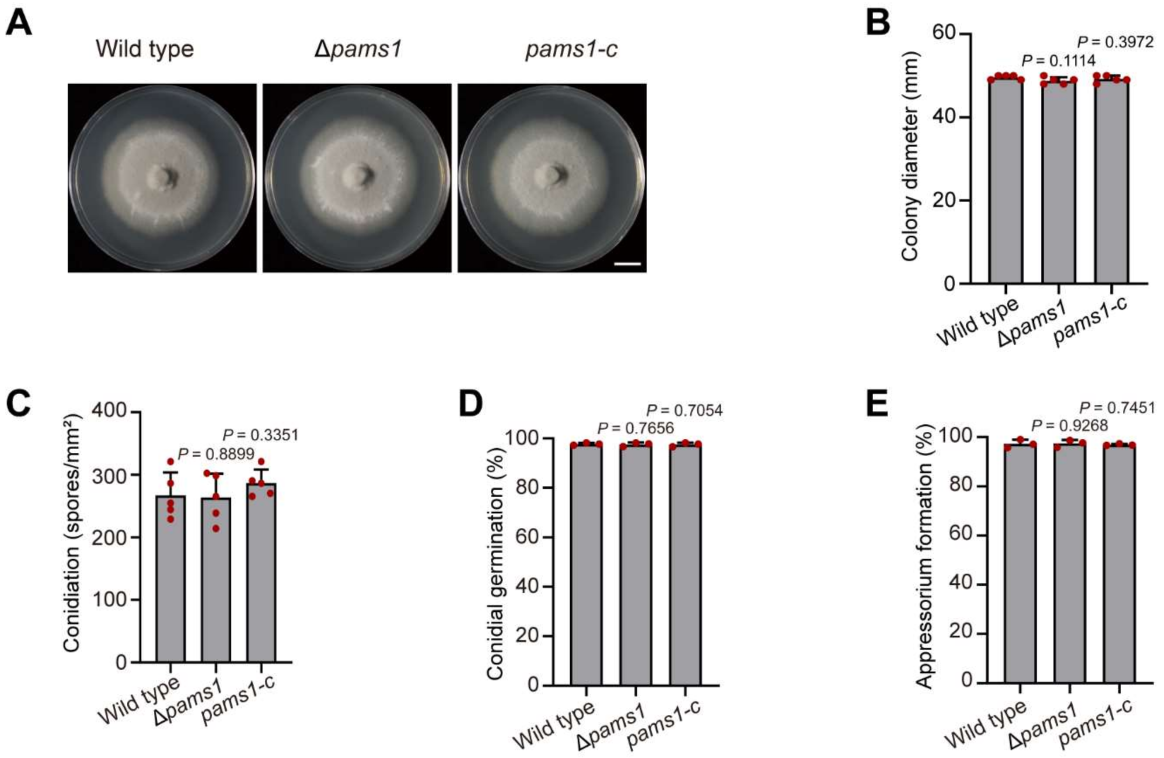

Supplementary FIGURE S2 Characteristics of Δ*pams1* in growth, conidiation, spore germination, and appressorium formation. (**A**) Micrographs showing colonies of the wild type, Δ*pams1* and complementation strain *pams1-c*. The strains were cultured on CM for 8 d. Scale bar, 10 mm. (**B**) Mycelial growth in diameter of the strains at 8 d in culture (n = 5). (**C**) Quantification of conidiation of strains (n = 5). (**D**) Spore germination rates of the strains on plastic coverslips at 4 hpi (n = 5). At least 200 spores were counted per replicate. (**E**) Appressorium formation rates of the strains on plastic coverslips at 24 hpi (n = 3). At least 200 spores were counted per replicate. Unpaired two-tailed Student's t-test was used for comparisons with the wild type control (**B**, **C**, **D**, **E**).

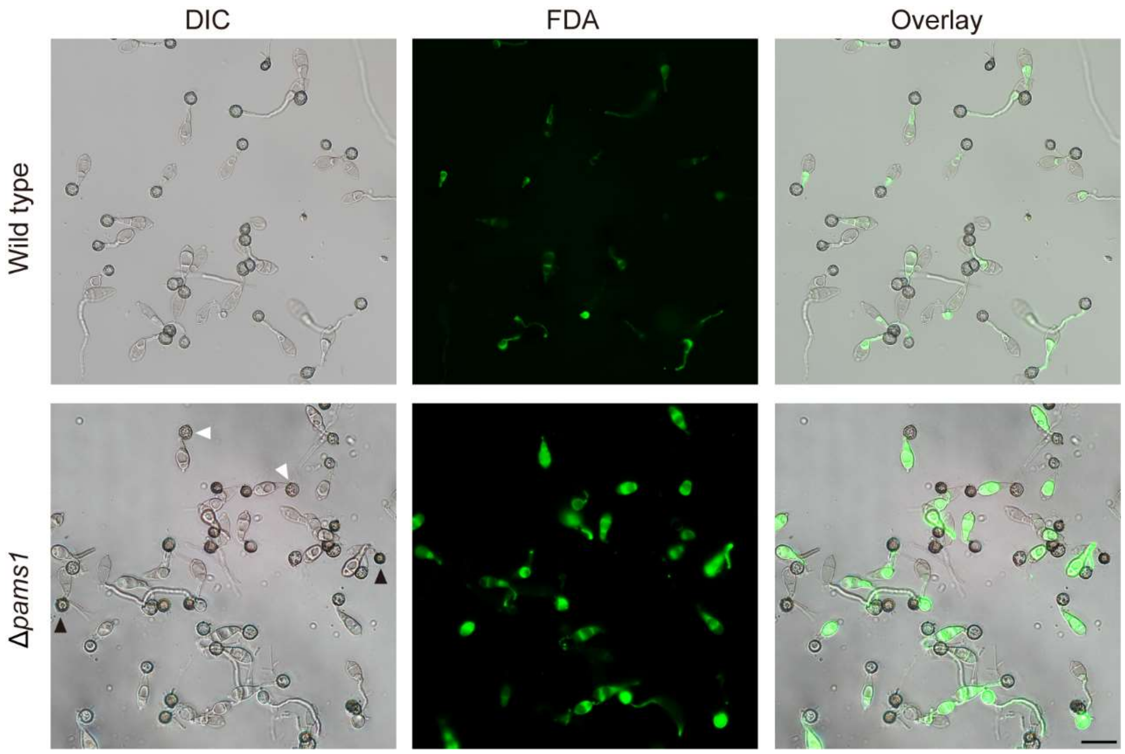

Supplementary FIGURE S3 The spore cells of Δ*pams1* that formed type II abnormal appressoria are viable at 24 hpi. (**A**) Appressoria of the wild type and Δ*pams1* stained by the dye fluorescein diacetate (FDA) at 24 hpi**.** The spore cells displaying green fluorescence indicate viable cells. Black arrows indicate type I abnormal appressoria, and white arrows indicate type II abnormal appressoria. Scale bar, 20 μm.

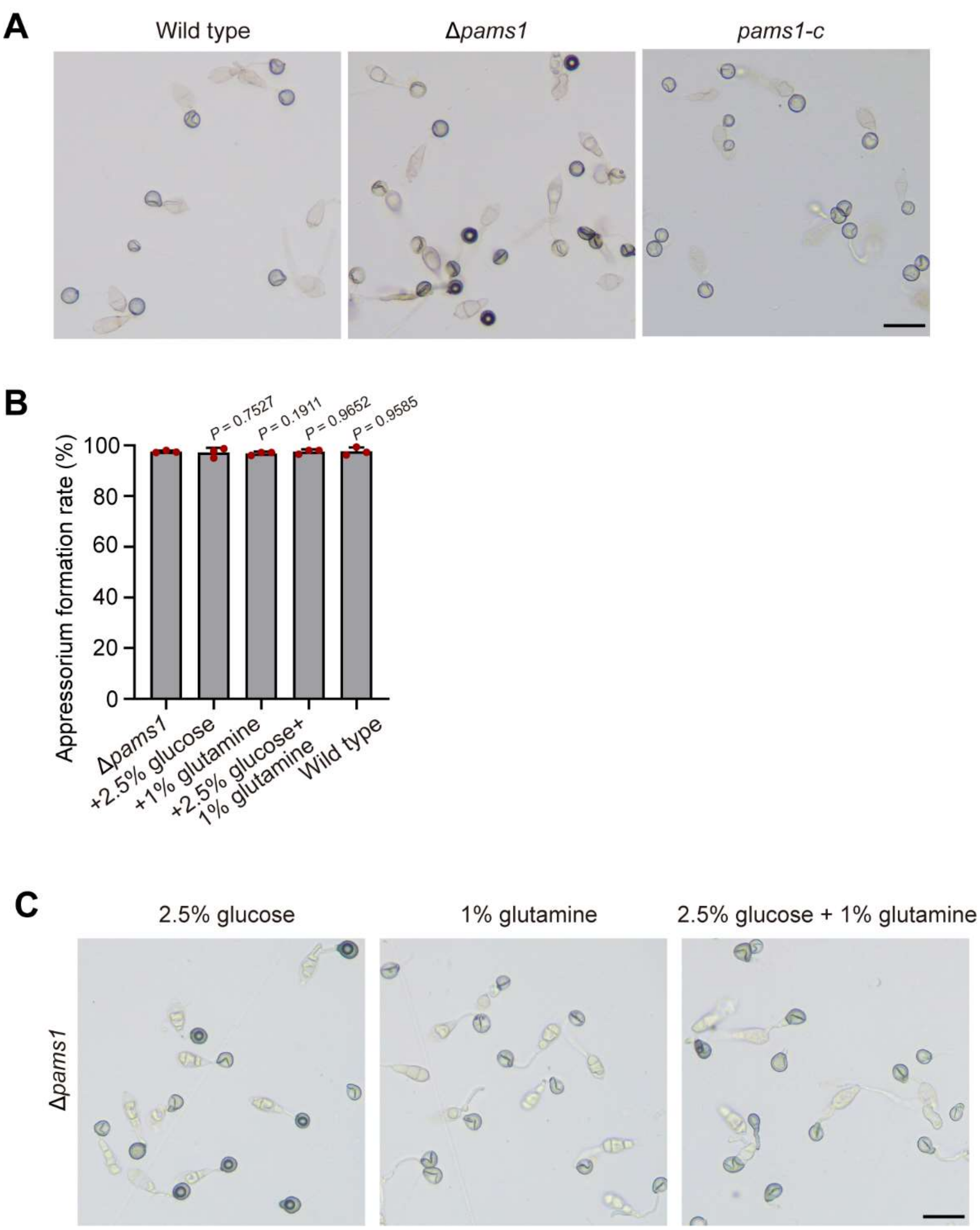

Supplementary FIGURE S4 Roles of Pams1 in appressorium cell walls. (**A**) Collapsed and plasmolyzed appressoria of the wild type, Δ*pams1*, and the complementation strain *pams1-c* in 1.5 M glycerol solution. (**B**) Addition of exogenous 2.5% glucose and/or 1% glutamine did not alter appressorium formation rate in Δ*pams1* (n = 3). At least 200 appressoria at 24 hpi were counted per replicate. Unpaired two-tailed Student's t-test was used for comparisons with the wild type. **P* < 0.05, ***P* < 0.01, ****P* < 0.001, *****P* < 0.0001. (**C**) Micrographs showing collapsed and plasmolyzed appressoria of Δ*pams1* formed after addition of 2.5% glucose, 1% glutamine, or both. Appressoria were immersed in 1.5 M glycerol solution for 5 minutes. (**A**, **C**) Scale bars, 20 μm.
